# Pneumatosis Intestinalis Developing in Association with Crohn’s Disease and Mimicking Gastrointestinal System Perforation

**Published:** 2011-01-01

**Authors:** Y Albayrak, S Aslan, A Kurt, Ü G Bayraktutan

**Affiliations:** 1 Department of General Surgery and Burn Unit, Erzurum Region Education and Research Hospital, Erzurum, Turkey; 2 Department of General Surgery, Erzurum Region Education and Research Hospital, Erzurum, Turkey; 3 Department of Pathology, Erzurum Region Education and Research Hospital, Erzurum, Turkey; 4 Department of Radiology, Erzurum Region Education and Research Hospital, Erzurum, Turkey

**Keywords:** Pneumatosis intestinalis, Crohn’s disease, Gastrointestinal perforation

Dear Editor,

Pneumatosis intestinalis (PI) is an uncommon disorder characterized by an accumulation of gas in the large or small bowel wall, and has been associated with a variety of disorders and procedures. Organ transplantation, bowel ischemia, inflammatory bowel disease, obstructive airway disease, infectious agents, and the administration of steroids or immunosuppressive agents are well known to predispose PI.[1] We present a patient with PI determined during exploration, referred to our hospital with a preliminary diagnosis of gastrointestinal system perforation.

**Fig. 1:**
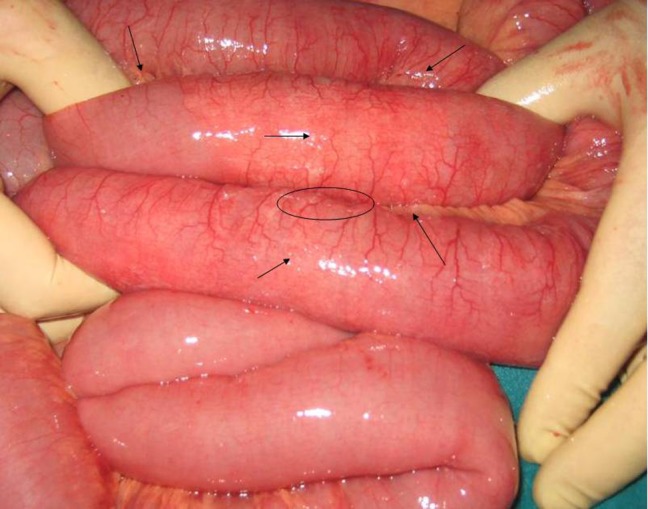
Intraoperative photograph: cystic lesions filled with gas and gas at the junction of mesentery and small bowel wall (arrows).

A 60-year-old male was sent to our hospital with abdominal pain and progressive abdominal distension for 2 days. Lung rear-front x-rays showed free air below the diaphragm. The emergency laparotomy showed serosal and mucosal thickening in the form of segmentary involvement in the ileum section of the intestines and pinhead-sized air-filled sacs in the intestinal serosa and mesentery tissue (Figure 1). No perforation foci were encountered in the stomach, or bowel. PI was therefore suspected under the circumstances; biopsies were taken from the intestinal serosa and mesentery and the laparotomy was concluded. In the pathological examination of the biopsies, connected air filled cysts positioned under the serosa were observed. Crohn’s disease was diagnosed as a result of the biopsies taken from the ulcers.

The pathogenesis of PI is not completely understood, but it is thought to be multifactorial.[2] Both bacterial and mechanical theories have been proposed, based largely on clinical entities known to be associated with PI.[2] In addition, steroids, as well as other immunosuppressants, are listed among the clinical variables related to the development of PI.[3] The most common symptoms found in patients with pneumatosis are diarrhea, bloody stools, abdominal pain, abdominal distention, constipation, and tenesmus.[4] Plain abdominal roentgenography is the most useful and easy way to ensure the diagnosis of PI.[4] The focus of treatment is almost entirely on the associated illness inciting PI. In PI associated with conditions in which surgical treatment has no role and where no other definitive treatment exists, excellent results have been reported with the use of inspired oxygen as a treatment.[5] The resolution of gas collections has been reported after inhalation of oxygen and after hyperbaric oxygen therapy.[6][7] Before performing emergency laparotomy on patients who complain of abdominal pain and exhibit free air in the abdomen, and who have PI or known causes of PI, computed tomography must be performed to eliminate PI.
